# Rebound Intracranial Hypertension

**DOI:** 10.1007/s11916-024-01231-9

**Published:** 2024-03-02

**Authors:** Simy K. Parikh

**Affiliations:** Department of Neurology, Jefferson Headache Center, Thomas Jefferson University Hospitals, Philadelphia, PA USA

**Keywords:** Rebound intracranial hypertension, Spontaneous intracranial hypertension, CSF hydrodynamics, Idiopathic intracranial hypertension, CSF leak

## Abstract

**Purpose of Review:**

Rebound intracranial hypertension (RIH) is a post-procedural treatment complication in patients with spontaneous intracranial hypotension (SIH) characterized by transient high-pressure headache symptoms. This article reviews the epidemiology, clinical features, risk factors, and treatment options for RIH.

**Recent Findings:**

This article discusses how changes in underlying venous pressure and craniospinal elastance can explain symptoms of RIH, idiopathic intracranial hypertension (IIH), and SIH.

**Summary:**

The pathophysiology of RIH provides a clue for how high and low intracranial pressure disorders, such as IIH and SIH, are connected on a shared spectrum.

## Introduction

Spontaneous intracranial hypotension (SIH) is a syndrome of intracranial hypotension that occurs from a spontaneous spinal CSF dural defect or spinal CSF venous fistula. Rebound intracranial hypertension (RIH) is a clinically important syndrome that occurs following procedural treatment of SIH with epidural blood patching (EBP), CSF venous fistula (CSFVF) embolization or ligation, or surgical dural repair. RIH typically manifests as a self-remitting frontally located supine headache associated with a post-procedural increase in opening pressure [1, 2, 3•].

## Epidemiology

Current evidence regarding RIH epidemiology is derived from a limited number of single-center studies. Available incidence analyses are subject to single-center biases, and there is variability in follow-up for RIH symptom development. Anecdotally, RIH is noted to be a common complication following spinal CSF leak closure [1, 2]. A large single-center retrospective analysis suggests an incidence of 27.4% [3•]. Similarly, knowledge about RIH incidence overall and RIH incidence specific to treatment approaches is limited. In the aforementioned retrospective analysis, RIH developed in 29.4% of patients who underwent surgical management of SIH and 21.4% of patients who underwent non-surgical procedural management [3•]. Another single-center retrospective analysis of patients undergoing minimally invasive surgery for dural closure for SIH suggests an incidence of RIH in 36% of patients evaluated on discharge from the procedure [4]. Data on RIH incidence following CSFVF embolization are also emerging. A single-center retrospective analysis of 100 consecutive patients treated with transvenous embolization for CSFVF reported that the incidence of RIH correlated with treatment response [5]. The study noted that 5.2% of patients with complete resolution, 29.7% with improvement but no resolution, and 60% with no improvement of SIH symptoms experienced RIH requiring pharmacological management at three months post-procedure [5].

RIH appears to affect individuals of various age groups, from children to the elderly, with an increased incidence among females in their 40 s [1, 3•, 6, 7]. It is worth noting that SIH itself has a slight female predominance and is most common in middle-aged individuals, particularly between the ages of 40 and 50 [8].

## Clinical Characteristics

Clinicians’ awareness of the clinical presentation of RIH allows for appropriate patient counseling, management, and reassurance [9•]. In addition, objective measures, such as papilledema and CSF opening pressure, are inconsistent or infrequently measured, making clinical presentation the keystone for RIH diagnosis [3•].

A retrospective analysis of 113 patients with SIH by Schievink et al. and a 9-patient case series by Kranz et al. assessed the clinical features of RIH [1, 3•]. In most cases of RIH, the headache location changes, with pain occurring frontally or peri-orbitally in RIH instead of suboccipitally, as in SIH [1, 3•]. While not explicitly analyzed in the above studies, other typical clinical features of RIH include worsening with recumbency and worsened symptoms in the morning, as opposed to symptoms of SIH, which are characteristically worsened with an upright position and best in the morning after prolonged overnight recumbency [1, 3•]. Symptoms associated with RIH include nausea, vomiting, and blurred vision. Transient papilledema is a rare complication of RIH [1, 3•, 6, 10, 11].

In most cases, symptoms of RIH develop soon after the procedural treatment of SIH and are temporary. Studies indicate that symptoms most commonly develop within the first 48–72 h after blood patching and can even occur during the treatment procedure [1, 3•]. A significant minority of patients (22%) can develop symptoms within the first 3–7 days following the procedure [3•]. RIH commonly resolves within 6 weeks, and nearly all cases (94%) resolve within 3 months [3•].

The diagnosis of RIH can be difficult as it may present similarly to other conditions such as refractory SIH, migraine, or idiopathic intracranial hypertension (IIH). Schievink et al. have suggested the following clinical features for RIH diagnosis: (1) reverse orthostatic headache different from the original SIH headache; (2) resolution of headache following administration of oral acetazolamide; and (3) not better accounted for by another cause of headache [3•].

Notably, there are exceptions to the common clinical features of RIH. Some patients with RIH have occipital head pain like that of SIH or develop new non-frontal head pain [1, 3•]. Similarly, some patients with SIH initially present with frontal head pain and do not experience pain location change with RIH [3•]. Less commonly, symptoms develop weeks to months after the procedure and, in rare instances, persist for up to 12 months or more [1, 3•]. In cases of atypical symptoms or refractory RIH, further diagnostic testing is necessary to differentiate RIH from other potential causes of intracranial hypertension [9•].

## Risk Factors

The risk of developing RIH is likely multifactorial. Body habitus or an underlying high-pressure disorder may increase the risk of RIH, although data on these factors are limited. Schievink et al. found that morbidly obese and super-obese patients with CSF-venous fistulas are at increased risk of developing RIH and papilledema [11]. Spontaneous decompression from underlying IIH is one suspected etiology of SIH; therefore, a prior history or clinical suspicion of IIH is also considered to be a risk factor for RIH [12, 13]. Transverse sinus stenosis is also found in IIH. A retrospective analysis of 113 patients with SIH by Schievink et al. explored transverse sinus stenosis, a normal anatomic variant, as a potential risk factor for RIH [3•]. RIH occurred in 54.8% of individuals with a complete signal gap in one transverse sinus or any involvement of both transverse sinuses and in 25.8% of individuals with focal narrowing in one transverse sinus as detected by pre-treatment MRV, while only occurring in 19.4% of individuals without any narrowing detected [3•].

Objective measures of SIH, such as findings on MRI brain imaging and opening pressure, are not predictive of RIH risk, although findings on spinal MRI may be predictive. Brain sag, while common, is not present in all cases of RIH [12]. Although one study found that individuals with a low OP (< 100 mm CSF) are more likely to develop RIH, other studies found that pre-procedural opening pressures can vary widely and even be within the normal range in patients who subsequently develop RIH [3•, 12, 14]. The presence of an extensive extradural CSF collection on MRI, suggestive of a fast-flow CSF leak, is correlated with an increased risk of RIH [3•].

It is unknown if specific treatment approaches contribute to an increased risk for RIH. The incidence of RIH occurring following autologous blood patching vs. autologous blood patching and fibrin glue has not been studied, and RIH is reported to occur at varying rates following both types of procedures [13–17]. Although in the author’s clinical experience, RIH occurs more commonly in individuals who undergo a large volume (> 20 cm^3^) epidural blood patch as opposed to a small volume epidural blood patch, one study found that epidural blood patch volumes have not been found to significantly differ in individuals who develop RIH and those who do not [14].

There may be other factors that contribute to the development of RIH. In the author’s opinion, factors such as SIH symptom duration prior to procedural treatment and epidural venous plexus dilation on spinal imaging should be considered in future studies assessing risk factors for RIH.

## Pathophysiology

There are varied theories on the pathophysiology of RIH. These suggest that RIH develops due to compensatory changes in CSF production, CSF reabsorption, cerebral venous outflow restriction, displacement of CSF by patching material, or a combination of these pathways [1, 3•, 6]. The following discussion on RIH pathophysiology considers similarities between SIH and IIH, specifically similarities in compensatory changes of venous flow and elastance of the craniospinal space, which are presumably also occurring in RIH.

**Fig. 1:**
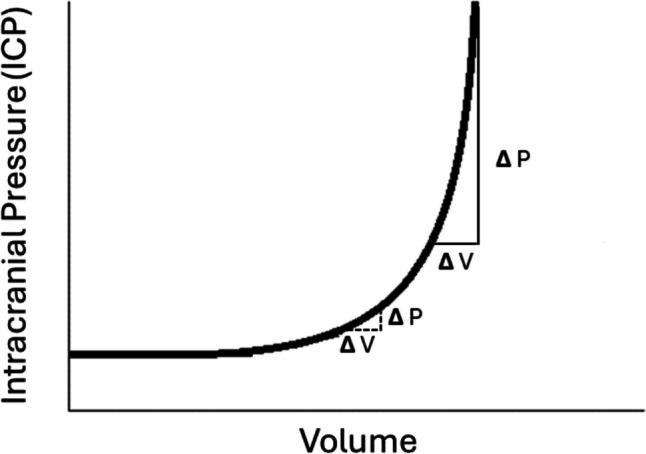
Hypothetical intracranial pressure–volume curve. As a system moves from a state of lower elastance (dotted line) to higher elastance (solid line), the same increase in volume results in higher intracranial pressure

Fundamentally, intradural venous sinus pressure is a key component of intracranial pressure equilibrium as per Davson’s equation of CSF absorption [18–21]. Venous pressure also influences craniospinal elastance. Elastance measures the pressure response of the intracranial system to a known change in volume. In a state of increased elastance, a little addition of volume can provoke a disproportionate increase in intracranial pressure (Fig. 1). Increased craniospinal elastance has been shown to occur in IIH and cases of compensated SIH and may exist in patients with RIH [22, 23•].

What causes this system of increased craniospinal elastance? One reason is a venous system that is reaching the limits of its compensatory abilities. In normal physiology, veins are capacitance vessels, accommodating large volumes of blood and acting as a buffer for changes in pressure and volume. They inherently have less elastance than arteries and can distend largely to accommodate changes in volume without resulting in significant pressure shifts, and vice versa. However, venous elastance is increased in an overcompensating venous system. This means that the normal craniospinal space reserve and ability to buffer changes in intracranial volume are diminished. Increased craniospinal elastance can lead to higher ICP, which is pathological in IIH and compensatory in SIH [22, 24].

Typically, in uncompensated SIH, it is hypothesized that there is actually low craniospinal elastance, which can directly cause low intracranial pressure itself or occur due to CSF volume loss [22, 25]. This theory is supported by lumbar infusion testing showing that the pressure–volume index (PVI), or the volume that is needed to be added to raise the pressure tenfold, is higher in patients with proven CSF leaks and in patients with a short duration of the disease [25, 26]. Lower craniospinal elastance has also been shown in patients with SIH without imaging signs of compensation for CSF volume loss [22].

However, in compensated SIH, craniospinal elastance increases. Two contributing factors to increased craniospinal elastance include (1) a compensatory increase in venous volume via the Monro–Kellie hypothesis and (2) a decrease in transcranial venous outflow. In SIH, the primary mechanism for compensating for the loss of CSF volume within the enclosed craniospinal space is the macro-veins within the spinal epidural space, which distend and increase in volume as per the homeostatic principles of the Monro–Kellie hypothesis [22]. This new baseline of added volume within the epidural venous plexus subsequently increases craniospinal elastance; it compromises the craniospinal venous system’s ability to accommodate subsequent increases in volume without significant craniospinal pressure increases [22]. In normal physiology, the internal jugular veins collapse in an upright position to maintain intracranial pressure equilibrium [19, 20, 27]. Patients with compensated SIH have a disproportionate decrease in internal jugular vein (IJV) outflow to that of total cerebral blood flow (tCBF); the reduction in venous outflow is speculated to help divert flow to the epidural, vertebral, and cerebral veins and serve as another compensatory mechanism for intracranial hypotension [22]. Consequently, patients with compensated SIH with high intracranial elastance are also noted to have higher opening pressures than those without increased intracranial elastance [18]. Other studies show that opening pressure increases with the length of the symptom duration in patients with SIH, presumably due to compensatory mechanisms [26, 28].

Like in SIH, in IIH, cerebral venous sinuses initially distend. In the case of IIH, initial venous distension occurs to accommodate for increases in abdominal and central venous pressure due to obesity or other undefined processes [29]. Simultaneously, venous distension reduces CSF absorption across the subarachnoid-venous sinus gradient, resulting in CSF accumulation and, ultimately, intracranial hypertension. Eventually, increased intracranial pressure on a stressed venous system results in venous sinus collapse and stenosis at focal weak points such as the transverse sinuses [29]. Like in SIH, in IIH, the venous sinuses have hypothetically overcompensated, resulting in a state of high craniospinal elastance where small changes in volume result in disproportionate changes in pressure.

Studies support the hypothesis that a state of high craniospinal elastance exists in IIH. Studies measuring the opening and closing opening pressures after CSF volume removal in patients with IIH have shown that IIH is associated with increased craniospinal elastance and decreased PVI and that increased craniospinal elastance is linearly correlated with increased opening pressure [24, 30]. Conversely, to assess SIH, researchers augmented pressure in patients with SIH via intrathecal normal saline [23•]. Although elastance measures in this study were not compared directly to opening pressures, the analysis showed that opening pressures were often normal as opposed to low, as one would expect in SIH [23•, 25]. Higher opening pressures and higher average elastance were significantly associated with cerebral venous sinus distension, pachymeningeal enhancement (micro-venous distension), and the presence of subdural collections in MRI brain imaging but not with signs of caudal brain displacement, further linking compensatory increased intracranial vascular volume with increased elastance [23•].

How does this understanding of craniospinal elastance inform us about the pathophysiology of RIH? In both disorders of IIH and compensated SIH, there seems to be a state of increased craniospinal elastance. In the case of RIH, the symptoms likely stem from the existence of such a state, in which the addition of volume leads to a disintegrating increase in intracranial pressure and clinical symptoms suggestive of a high-pressure headache. Current evidence cannot yet answer the question of why only some patients with SIH develop RIH while others do not. Hypothetically, it is those patients with high craniospinal elastance, such as those with compensated SIH or underlying IIH, who would be predisposed to developing RIH. A study by Schievink et al. (2019) supports the link between changes in venous physiology and RIH, finding that transverse sinus narrowing is associated with a higher chance of RIH [3•]. Using MRV, the study found that a fourth of those with focal narrowing in one transverse sinus and half of those with a complete signal gap in one transverse sinus or any involvement of both transverse sinuses developed RIH [3•].

Variability in RIH onset could occur from variability in an individual’s reserve of the craniospinal elastance system as a whole, with symptoms occurring at the point in which volume shifts resulting from the procedure and/or subsequent healing process and ensuing fibrosis overwhelm an individual’s buffering reserve. Another explanation for variability in onset could be that the craniospinal elastance system is also affected by variations in the compliance of the dura itself, which affects CSF outflow resistance. Those with a high complaint dura and low CSF outflow resistance may not, or to a lesser degree, experience RIH. Others who have developed adhesions, scarring, and/or fibrosis as part of natural or induced dural healing, which results in decreased CSF compliance and increased CSF outflow resistance, may be more vulnerable to RIH [25].

Exploring RIH pathophysiology may also provide insight into the pathophysiology of other primary headache disorders. It is reasonable to theorize that increased craniospinal elastance, potentially mediated by changes in venous physiology, exists in other primary headache disorders [31]. A low reserve to compensate for changes in pressure or volume could explain some phenomena seen in primary headache disorders that otherwise do not have an anatomical explanation, such as primary headache disorders with pain resulting from increases in intrabdominal pressure through Valsalva maneuvers or cough, from increases in cardiovascular output (e.g., through exertion), and from barometric pressure changes.

## Treatment

RIH management focuses on improving the impact of symptoms on quality of life. In many cases, RIH can be conservatively or pharmacologically managed; lumbar puncture and CSF drainage are only recommended for intolerable symptoms or in cases of diagnostic uncertainty [1, 3•, 9•]. Conservative measures include positioning the head at a 20–30° elevation, which has been shown to reduce ICP [3•, 21]. If conservative measures are not sufficient, additional interventions include the use of oral pharmacological agents, such as acetazolamide [3•]. Acetazolamide is a carbonic anhydrase inhibitor that reduces ion and water transport across the choroid plexus, thereby reducing CSF production and lowering intracranial pressure [32]. It has been utilized in managing other forms of intracranial hypertension and may have potential utility in treating RIH. However, the use of acetazolamide in treating RIH has not extensively been studied, and specific literature on this topic is limited. There is no evidence regarding the effective dosing of acetazolamide for RIH. Commonly, the starting dose emulates the dose used for IIH, which is 250 to 500 mg twice a day, with titration as needed; acetazolamide can be taken up to four times/day and has an acceptable safety profile at doses up to 4000 mg/day [1, 3•, 33]. Schievink et al. describe a regimen of acetazolamide at a dose of 500 mg by mouth taken twice or three times a day, up to doses of 3000 mg daily, to manage symptoms [3•]. If acetazolamide is not tolerated, other carbonic anhydrase inhibitors, such as methazolamide and topiramate, or diuretics, such as furosemide or hydrochlorothiazide, have been anecdotally used for treatment [1, 3•, 9•]. RIH is self-remitting in most cases, allowing for treatment discontinuation at symptom resolution [9•]. In cases of severe RIH, CSF drainage via lumbar puncture may be needed [1, 3•].

The emergence of RIH indicates an intracranial pressure shift; however, its true impact on dural healing, whether positive or negative, is unclear. RIH following SIH management is viewed as a positive prognostic sign as it suggests successful treatment of the CSF leak [3•]. As such, RIH is typically managed with conservative measures and only treated with pharmacological agents to manage symptoms rather than reduce the chance of CSF leak recurrence [1, 3•, 9•]. However, in cases where SIH is suspected to occur from spontaneous decompression due to underlying IIH, more aggressive post-procedural intracranial pressure management could reduce the risk of leak recurrence. Cranial CSF leaks resulting from spontaneous decompression due to underlying IIH do have a higher rate of failure after repair than those of traumatic origin [34]. In such cases, aggressive post-procedural intracranial pressure management of elevated intracranial pressure has been shown to have statistical significance in improving procedural success [35]. Interestingly, another consideration is using a protocol for pre-treatment with acetazolamide [36]. Ferrante et al. studied the benefit of pre-medication with acetazolamide at a dosage of 250 mg twice a day at 18 and 6 h prior to a large-volume untargeted lumbar epidural blood patch in the Trendelenburg position. With the caveat that follow-up time was not noted, the authors documented that no patient developed RIH with this pre-medication regimen [36]. While not all individuals treated for SIH develop RIH, having a quickly implementable treatment plan for patients who develop RIH symptoms or implementing a pre-medication plan for those who have risk factors for an IIH etiology or obvious venous compensatory signs could improve the prognosis.

While acetazolamide has demonstrated efficacy in reducing intracranial pressure in other forms of intracranial hypertension, the evidence for it or other specific treatments for RIH is not well established. Individualized treatment decisions should consider patient characteristics and responses to other interventions, and further research is needed to establish a treatment course for managing or preventing RIH.

## Conclusion

Rebound intracranial hypertension is an important clinical condition presenting as a high-pressure headache after the treatment of SIH. Formal diagnostic criteria for RIH can help standardize the study of RIH. Large-scale multi-institutional studies assessing the development of RIH at specific time points post-procedure would be valuable in providing more precise estimates of its epidemiology. While the pathophysiology of RIH is speculative, it could reflect a high elastance system similar to compensated SIH and IIH. Further understanding of RIH pathophysiology can subsequently help identify risk factors for developing RIH. Current treatment of RIH involves conservative management and carbonic anhydrase inhibitors. Further insights into RIH can help determine optimal pre-treatment regimens and post-treatment symptom management.

## Data Availability

No datasets were generated or analyzed during the current study.
